# Epigenetic silencing of *UBXN8* contributes to leukemogenesis in t(8;21) acute myeloid leukemia

**DOI:** 10.1038/s12276-021-00695-8

**Published:** 2021-12-17

**Authors:** Erna Yang, Wei Guan, Desheng Gong, Jieying Li, Caixia Han, Juan Zhang, Hong Wang, Synat Kang, Xuefeng Gao, Yonghui Li, Li Yu

**Affiliations:** 1 Department of Hematology and Oncology, International Cancer Center, Shenzhen Key Laboratory, Shenzhen University General Hospital, Shenzhen University Clinical Medical Academy, Shenzhen University Health Science Center, Xueyuan AVE 1098, 518000 Shenzhen, China; 2 Department of Hematology, Chinese PLA General Hospital, 100853 Beijing, China; 3 School of Medicine, Nankai University, 94 Weijin Road, 300071 Tianjin, China; 4 Central Laboratory, Shenzhen University General Hospital, Xueyuan AVE 1098, Nanshan District, 518000 Shenzhen, Guangdong People’s Republic of China

**Keywords:** Acute myeloid leukaemia, Tumour heterogeneity

## Abstract

The formation of the RUNX1-RUNX1T1 fusion protein, resulting from the t(8;21) translocation, is considered to be one of the initiating events of t(8;21) acute myeloid leukemia (AML). However, the mechanisms of the oncogenic mechanism of RUNX1-RUNX1T1 remain unclear. In this study, we found that RUNX1-RUNX1T1 triggers the heterochromatic silencing of *UBXN8* by recognizing the RUNX1-binding sites and recruiting chromatin-remodeling enzymes to the *UBXN8* promoter region. Decitabine, a specific inhibitor of DNA methylation, upregulated the expression of *UBXN8* in RUNX1-RUNX1T1^+^ AML cell lines. Overexpression of *UBXN8* inhibited the proliferation and colony-forming ability of and promoted cell cycle arrest in t(8;21) AML cell lines. Enhancing *UBXN8* levels can significantly inhibit tumor proliferation and promote the differentiation of RUNX1-RUNX1T1^+^ cells in vivo. In conclusion, our results indicated that epigenetic silencing of *UBXN8* via methylation of its promoter region mediated by the RUNX1-RUNX1T1 fusion protein contributes to the leukemogenesis of t(8;21) AML and that *UBXN8* targeting may be a potential therapeutic strategy for t(8;21) AML.

## Cancer: Gene modifications promote leukemia progression

The protein byproduct of a common chromosomal rearrangement in acute myeloid leukemia (AML) promotes cancerous growth by inhibiting a tumor suppressor protein. AML cells that exhibit an abnormal coupling of segments from chromosomes 8 and 21 produce a fusion protein known as RUNX1-RUNX1T1. Erna Yang of Shenzhen University General Hospital, China, and colleagues have identified a mechanism by which this protein promotes AML progression. Experiments with AML cell lines showed that RUNX1-RUNX1T1 recruits DNA-binding proteins that introduce numerous chemical modifications to the *UBXN8* gene, which inhibit production of the tumor suppressor protein it codes for. Drugs that block these modifications and stimulate *UBXN8* activity also arrest tumor cell division. Experiments in mice confirmed that enhanced *UBXN8* expression offers a defense against AML progression, suggesting a promising therapeutic approach.

## Introduction

The t(8;21)(q22;q22) translocation is one of the most common chromosomal aberrations in acute myeloid leukemia (AML), and this abnormality is predominantly present in the AML French-American-British (FAB)-M2 subtype^1^. Since the discovery of the RUNX1-RUNX1T1 fusion protein, numerous studies have revealed that t(8;21) AML is a highly heterogeneous disease from a biological and a clinical point of view^2^. The RUNX1-RUNX1T1 fusion protein is considered to be one of the t(8;21) AML initiating events and represents the unique molecular characteristics of t(8;21) AML. It is well established that genetic aberrations play a key role in the diagnosis, treatment, and prognosis of AML, but nearly 50% of AML patients have a normal karyotype, and many of them carry no mutations. Recent genome-wide studies identified unique DNA methylation signatures for subsets of AML patients, indicating that t(8;21) AML patients have a unique methylation pattern and that such an abnormal methylation pattern leads to aberrant expression of the gene, which plays an important role in the occurrence and development of this disease^3,4^.

*UBXN8*, also known as *UBXD6* or *REP-8*, encodes a member of the ubiquitin regulatory X (UBX) protein family^5^, the largest known group of p97 cofactors, which is a transmembrane protein localized in the endoplasmic reticulum (ER) membrane. UBXN8 tethers p97, a versatile ATPase complex, to the ER membrane *via* its UBX domain, and the association of this cofactor with p97 facilitates the efficient ER-associated degradation (ERAD) of misfolded proteins^6^. The available evidence strongly suggests that several members of the UBX protein family regulate processes associated with oncogenesis, such as cell proliferation and apoptosis^7–9^. Among the UBX protein family members, UBXN8 was identified as a new tumor suppressor candidate that functions in a TP53-dependent manner in hepatocellular carcinoma (HCC)^10^.

In this study, we used methylC-capture sequencing (MCC-Seq)^11^ to detect the unique methylation pattern of RUNX1-RUNX1T1^+^ cell lines and found a specific promoter hypermethylation pattern of the *UBXN8* gene in the RUNX1-RUNX1T1^+^ cell line. This study also showed that the *UBXN8* gene was specifically downregulated in RUNX1-RUNX1T1^+^ leukemia cell lines. It revealed that the fusion protein RUNX1-RUNX1T1 could directly bind to the RUNX1-binding sites along the promoter region of the *UBXN8* gene and recruit chromatin-remodeling enzymes, thereby leading to the methylation of CpG islands and the epigenetic silencing of the *UBXN8* gene. In addition, decitabine, a specific inhibitor of DNA methylation, upregulated the expression of *UBXN8* in RUNX1-RUNX1T1^+^ leukemia cell lines. Upregulation of UBXN8 expression significantly inhibited cell proliferation and colony formation, induced G1 arrest, and promoted cell differentiation in SKNO-1 cells. In vivo experiments demonstrated that upregulation of *UBXN8* significantly slowed cell proliferation and promoted cell differentiation in xenograft mice subcutaneously inoculated with SKNO-1 cells. Our data suggest that the epigenetic silencing of *UBXN8* mediated by the RUNX1-RUNX1T1 oncoprotein contributes to the leukemogenesis of t(8;21) AML and that *UBXN8* plays a tumor suppressor role in t(8;21) AML; thus, enhancing UBXN8 expression may be a potential treatment option for t(8;21) AML.

## Material and methods

### Cell lines and transfection

The SKNO-1, SKNO-siAE, U-937AE, U-937, Kasumi-1, HL-60, MV4-11, and THP-1 cell lines were maintained in RPMI 1640 medium supplemented with 10% fetal calf serum (HyClone, Logan, UT, USA), penicillin (10^7^ U/L) and streptomycin (10 mg/L). Human embryonic kidney (HEK) 293T cells were cultured in DMEM supplemented with 10% fetal calf serum (HyClone, GE Healthcare, Logan, UT, USA), penicillin (10^7^ U/L) and streptomycin (10 mg/L). All cells were kept in a humidified incubator at 5% CO_2_ and 37 °C. pRRLcPPT.hPGK, a lentiviral vector encoding the previously described^12^ siAGF1 oligonucleotides (Additional file 1. Table of primers), which were designed against the RUNX1-RUNX1T1 mRNA fusion site, was used to silence RUNX1-RUNX1T1 in SKNO-1 cells^12,13^ and produce SKNO-siAE cell lines. The U-937AE clone was obtained by subcloning HA-tagged RUNX1-RUNX1T1 cDNA into a vector carrying the Zn ^2+^-inducible mouse MT-1 promoter and electroporating the vector into U-937 wild-type cells. ZnSO_4_ (100 μM, 16 h) treatment was used to induce RUNX1-RUNX1T1 expression in U-937AE cells^14^. SKNO-1, SKNO-1-siAE, U-937AE and U-937 cell lines were kind gifts from Prof. Clara Nervi (Department of Medical and Surgical Sciences and Biotechnologies, University of Roma “La Sapienza”, Corso della Repubblica 79, Latina I-04100. E-mail: clara.nervi@uniroma1.it). HL-60, MV4-11, THP-1, Kasumi-1-1, and HEK293T cells were obtained from the Hematology Laboratory of Chinese PLA General Hospital. All human cell lines were authenticated with STR profiling within the last three years, and all experiments were performed with mycoplasma-free cells. The *UBXN8* lentiviral expression vector Lenti-UBXN8 and control lentiviral vector were purchased from GeneChem Co., Ltd. (Shanghai, China) and used for the ectopic induction of *UBXN8* in SKNO-1 cells to generate Lenti-UBXN8 cells. After transduction with Lenti-UBXN8 or control lentiviral vectors at an MOI of 100, the SKNO-1 cells were centrifuged at 1000 × *g* for 3 h in the presence of 5 ng/mL polybrene. Subsequently, the medium was changed to puromycin-containing medium, and cells were cultured for 48 h to select for stably transduced cells. Puromycin-resistant colonies were further selected for 3 weeks and then expanded and confirmed by flow cytometry. Treatment with decitabine (Sigma–Aldrich, Saint Louis, MO, USA) was performed at a concentration of 1.0 μM for 72 h.

### Clinical samples

Normal mononuclear cells were isolated from bone marrow from consenting healthy donors. Informed consent was obtained in accordance with the principles of the Helsinki Declaration, and the protocol was approved by the Human Subject Ethics Committee of the Chinese PLA General Hospital.

### RNA extraction and analysis

Total RNA was extracted using TRIzol Reagent (Invitrogen Carlsbad, CA, USA). cDNA was obtained by reverse transcription. The expression of *UBXN8* mRNA was quantified by SYBR Green real-time quantitative polymerase chain reaction (qRT-PCR) analysis (Takara Biomedical Technology Co., Ltd., Beijing, China) on an Mx3000P LightCycler (Stratagene, La Jolla, CA, USA). Gene expression was analyzed by the 2^−ΔΔCt^ method^15^ using *GAPDH* as an internal control. The primer sequences are shown in Additional file 1. Table of primers.

### Western blot analysis

Western blot analysis was performed as described elsewhere^14^. Recombinant human anti-UBXN8 was purchased from Abcam (ab159924; Cambridge, UK). Rabbit anti-human β-actin (ab179467; Cambridge, UK) was used as a control.

### MCC-Seq assay

The MCC-Seq assay requires the preparation of qualified DNA samples and a whole-genomic methylation sequencing library, bisulfite conversion, target bisulfite-converted DNA area capture and enrichment using the custom probe, and amplification. This process was performed using the novel SeqCap Epi probe design platform of Roche NimbleGen, which is a fixed epigenome-wide design that allows the interrogation of more than 5.5 million methylation sites per sample and enables the capture of double-stranded targets regardless of their methylated state via high-density tiling of probes. Each capture was sequenced in a single lane of the 125-bp paired-end Illumina HiSeq 2500 System^11^. Adapters were cut off from the raw reads and then trimmed for quality (phred33 ≥ 30) and length (n ≥ 50) using Perl script (FQ_clean_v2.0.pl, parameters: -q 5 -G 20 -L 50 -r 0.5 -N 0.1 -P 33). The filtered reads were aligned to the hg19 human reference genome using BSMAP (v 2.73, parameter: -v 0.1 -g 1 -p 8 -R -u)^16^. Methylation calls were extracted based on the unique sequences. In all subsequent analyses, a sequence depth of ≥5X was used to detect the methylation level of CpG islands. Metilene (v 0.2-6)^17^ was employed to identify maximal between-group methylation differences in genomic regions of minimum length in combination with the Mann–Whitney *U*-test. The results were further refined by selecting DMRs meeting the following criteria: (1) minimum of five CpG sites, (2) maximum CpG distance (default: 300), (3) minimum CpGs, 5, (4) minimum methylation difference (D- absolute value) ≥0.2, and (5) adjusted *p*-value < 0.05.

### DNA extraction and bisulfite-sequencing PCR (BSP)

Genomic DNA isolation from the cell lines and healthy donors was performed using the Wizard® Genomic DNA Purification Kit (Promega Corp., Madison, WI, USA), and bisulfite DNA conversion was performed using the Epi-Tect-Bisulfite Kit (Qiagen, Hilden, Germany) according to the manufacturer’s instructions. The primer sequences for BSP are shown in Additional file 1. Table of primers. For BSP, the amplified PCR products were purified and then cloned into the pGEM-T vector (Promega) system. PCR of individual bacterial colonies was performed using vector-specific primers, and the products were sequenced for analysis of DNA methylation status.

### Transactivation assays

DNA fragments were synthesized by Sangon Biotech (Shanghai, China) and inserted into the pGL3-LUC reporter vector (Promega). Approximately 2 × 10^5^ HEK293T cells were plated in 24-well plates and transiently cotransfected with 10, 50, or 100 ng of the pcDNA3.0 vector with or without HA-tagged RUNX1/RUNX1T1 cDNA^12^ and 400 ng of the LUC reporter construct. Cotransfection with 10 ng of the pRL-TK Renilla luciferase reporter vector (Promega) was used as an internal control. Lipofectamine™ 2000 Transfection Reagent (Invitrogen) was used for transfection. Cells were harvested 48 h after transfection and assayed using the dual-luciferase assay (Promega) according to the manufacturer’s instructions.

### Chromatin immunoprecipitation (ChIP) assays

ChIP assays were performed using the Simple ChIP^®^ Plus Enzymatic Chromatin IP Kit (9005#; Cell Signaling Technology, Danvers, MA, USA) according to the manufacturer’s instructions. Briefly, cross-linked chromatin from approximately 4 × 10^8^ SKNO-1 cells or 4 × 10^8^ SKNO-siAE cells was prepared and fragmented to an average size of approximately 200 bp by 35 cycles of sonication (30 s each) in 1.5 mL tubes using a Bioruptor UCD-200 sonicator (Diagenode, Sparta, NJ, USA). After sonication, chromatin was immunoprecipitated overnight with 5 µg of antibodies against RUNX1 (ab23980; Abcam), RUNX1T1 (ab195329; Abcam), DNMT3A (ab2850; Abcam), DNMT3B (ab2851; Abcam), or DNMT1 (ab92314; Abcam). Normal mouse immunoglobulin G (IgG) served as a negative control. DNA fragments obtained without antibody were used as the input controls. The *UBXN8* genomic upstream region surrounding the RUNX1-binding site and unrelated negative control region were amplified by SYBR Green qRT-PCR analysis (Takara) on an Mx3000P LightCycler (Stratagene). The primer sequences are shown in Additional file 1. Table of primers. The expression was analyzed by the 2^−ΔΔCt^ method and reported as the value relative to that of the input controls. *GAPDH* served as an internal control accounting for nonspecific precipitated sequences.

### Cell proliferation, differentiation, cycle, and colony formation assays

Cell proliferation was measured with the Cell Counting Kit (CCK-8) (Dojindo, Kumamoto, Japan) according to the manufacturer’s instructions. Briefly, 5 × 10^3^ cells were seeded into 96-well plates, and then 10 μl of CCK-8 solution was added followed by a 3-h incubation at 37 °C in a 5% CO_2_ atmosphere before detecting the absorbance of each well at a wavelength of 450 nm. For the colony formation assays, cells were seeded in a methylcellulose H4230 culture system (Stem Cell Technologies, Vancouver, BC, Canada) at a density of 500 cells per mL. After incubation for 10 days at 37 °C in a 5% CO_2_ atmosphere, localized clusters containing > 50 cells and showing morphological hematopoietic characteristics were counted as colonies. Cell cycle analysis was performed using the C543 cell cycle assay kit (Dojindo). Briefly, 5 × 10^6^ cells were collected for each sample and washed twice with PBS. Then, the cells were stained with a propidium iodide (PI) solution for 30 min in the dark and analyzed by flow cytometry. For cell differentiation, samples were stained with CD11b (301350; BioLegend, San Diego, CA, USA) and analyzed by flow cytometry. All experiments were performed in triplicate.

### Murine models

For the animal study, female BALB/c nude mice (6–8 weeks-old, 18–20 g body weight) were bred and maintained in a pathogen-free environment in the Laboratory Animal Center of Chinese PLA General Hospital. All BALB/c nude mice were treated in strict compliance with principles of the care and use of laboratory animals established by the Laboratory Animal Center of Chinese PLA General Hospital. The xenograft mouse model was established by implanting 2 × 10^7^ cells/100 μl/mouse in PBS via subcutaneous injection into the right flank. Tumor volumes were calculated using the following equation: V (in mm^3^) = *A* × *B*^2^/2^12^, where *A* is the largest diameter and *B* is the perpendicular diameter. The mice were killed by CO_2_ inhalation at day 23 after cell injection. For histological analysis, sections of the tumor tissues were prepared and stained following standard protocols as previously described^18^. Immunohistochemistry was performed using an anti-CD11b antibody (ab133357; Abcam) on an automated immunostainer (Ventana Medical Systems Inc., Tucson, AZ, USA) in accordance with the company´s protocols for open procedures with some minor modifications.

### Bioinformatics and statistical analysis

We found clinical samples from the public databases BloodSpot (http://servers.binf.ku.dk/bloodspot/?gene=UBXN8&dataset=MERGED_AML) and Gene Expression Omnibus (GSE13159). SPSS 13.0 software (SPSS Inc., Chicago, IL, USA) was used to process the data. All results are expressed as the means ± standard deviation (SD) from three separate experiments. Student’s *t*-test was used to determine the statistical significance of experimental results, and a *P*-value of ≤0.05 was considered statistically significant.

## Results

### *UBXN8* expression is downregulated in RUNX1-RUNX1T1^+^ leukemia cells

**Fig. 1:**
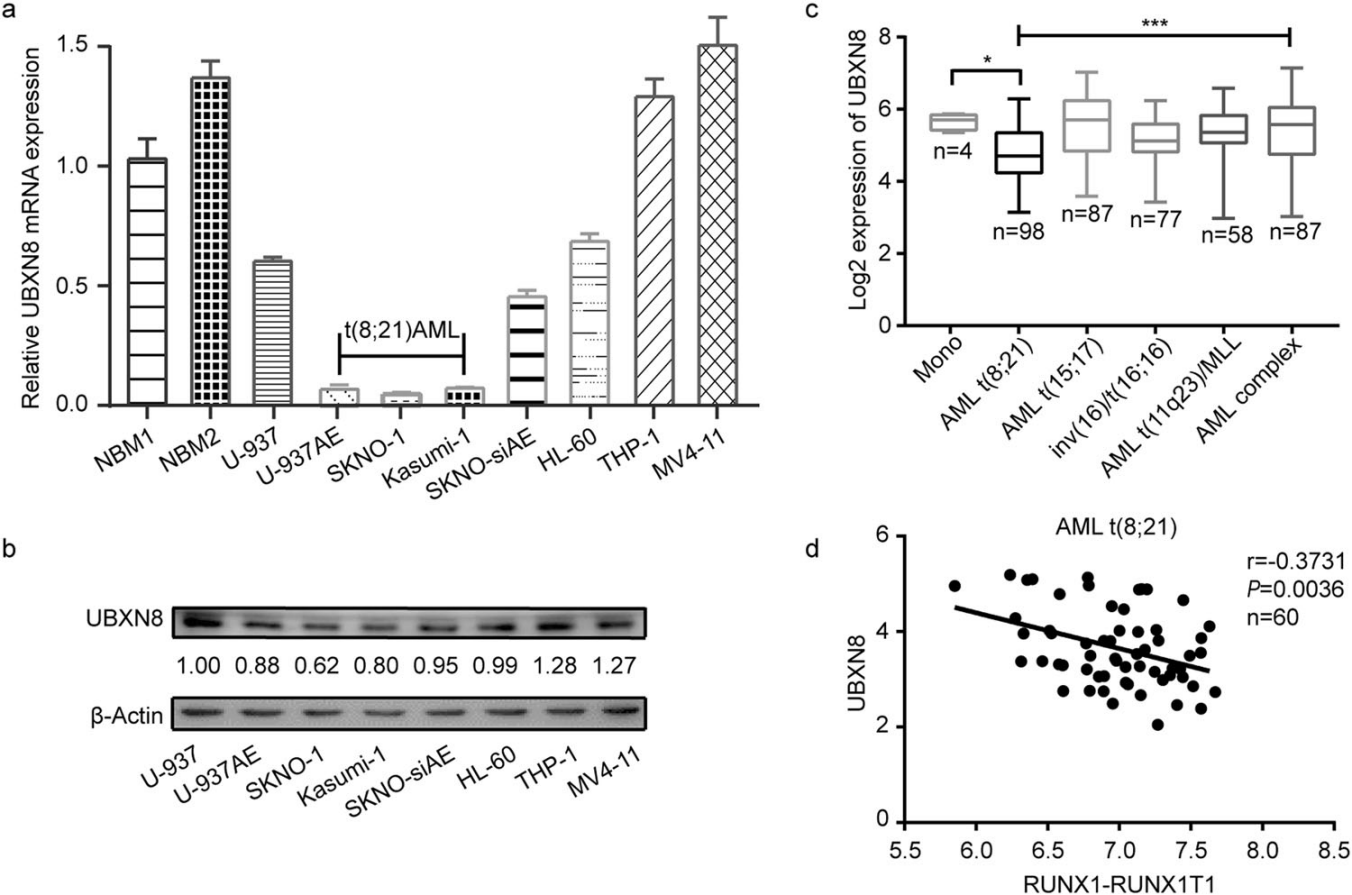
*UBXN8* expression is selectively downregulated in RUNX1-RUNX1T1^+^ AML cell lines. **a** *UBXN8* expression was downregulated in RUNX1-RUNX1T1^+^ cell lines (U-937AE, SKNO-1 and Kasumi-1 cell lines) compared to monocytes isolated from the two sets of normal bone marrow (NBM) and RUNX1-RUNX1T1^-^ cell lines (U-937, SKNO-siAE, HL-60, THP-1 and MV4-11). Error bars indicate the standard deviation (SD) from three different assays. **b** UBXN8 protein expression in AML cell lines was measured by Western blotting. **c** Expression of *UBXN8* in monocytes isolated from NBM and different AML subtypes from the BloodSpot database was analyzed. *UBXN8* expression was significantly downregulated in t(8;21) AML compared with normal bone marrow monocytes and other AML subtypes. **d** *UBXN8* mRNA expression was plotted against that of *RUNX1-RUNX1T1* in t(8;21) AML (*n* = 60) (GSE13159). The mRNA expression of *UBXN8* versus *RUNX1-RUNX1T1* showed a negative relationship. (^*^P < 0.05, ^***^P < 0.001).

Based on an initial observation of *UBXN8* expression in AML, we quantified the mRNA expression levels of *UBXN8* in two sets of NBM (normal bone marrow) from healthy donors and eight AML cell lines (U-937, U-937AE, SKNO-1, Kasumi-1, SKNO-siAE, HL-60, THP-1 and MV4-11). Notably, the mRNA expression of *UBXN8* was robustly downregulated in RUNX1-RUNX1T1^+^ cells (U-937AE treated with 100 μM ZnSO_4_ for 16 h, SKNO-1 and Kasumi-1) compared to the two sets of NBM and RUNX1-RUNX1T1^-^ cells (THP-1, MV4-11, U-937, HL-60 and SKNO-siAE) (Fig. 1a). This trend of UBXN8 expression was also confirmed at the protein level by Western blot analysis of the same eight AML cell lines (Fig. 1b). These results were subsequently validated in the public database BloodSpot, which included 4 normal monocyte lines, 98 t(8;21) AML samples, 87 t(15;17) AML samples, 77 inv (16)/t(16;16) AML samples, 58 t(11q23)/MLL samples, and 87 complex aberrant karyotype AML samples, revealing a significant downregulation of *UBXN8* expression in t(8;21) AML compared to the other subtypes of AML and healthy monocytes (Fig. 1c). More interestingly and notably, from the published GEO microarray dataset (GSE13159), we also found that the expression of *UBXN8* had a markedly negative relationship with the expression of RUNX1-RUNX1T1 in t(8;21) AML (Fig. 1d). Overall, these results indicated that *UBXN8* expression is selectively downregulated in RUNX1-RUNX1T1^+^ leukemia cells, suggesting that downregulated *UBXN8* expression is controlled by RUNX1-RUNX1T1.

### Epigenetic silencing of *UBXN8* in RUNX1-RUNX1T1^+^ cell lines by DNA methylation in the promoter region

**Fig. 2:**
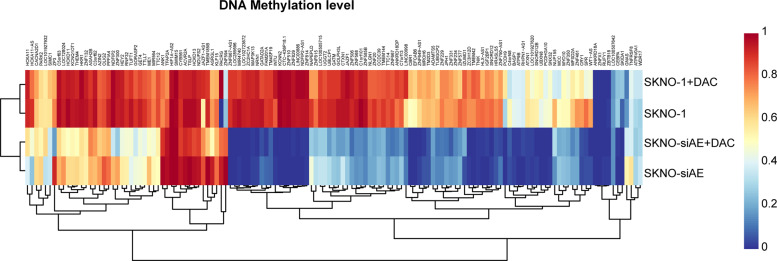
Altered DNA methylation status in RUNX1-RUNX1T1^+/−^ cell lines. The DNA methylation status in SKNO-1 and SKNO-siAE cells was changed in a large number of genes after treatment with 5-aza-2 deoxycytidine. In addition, the *UBXN8* gene was one of genes with an altered DNA methylation status.

**Fig. 3:**
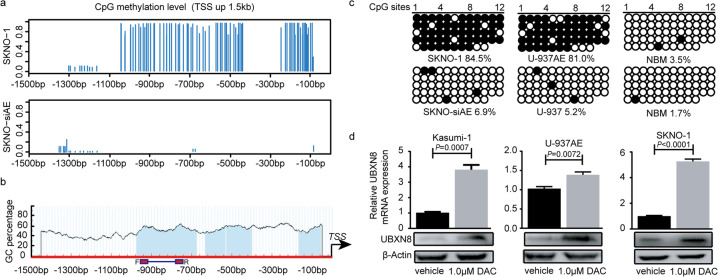
The *UBXN8* promoter region is highly methylated in RUNX1-RUNX1T1^+^ cell lines. **a** Methylation level at the *UBXN8* gene promoter region (–1500 to –1 bp, TSS up 1.5 kb) as analyzed by MCC-Seq was obviously higher in SKNO-1 cells than in SKNO-siAE cells. **b** Schematic representation of the distribution of the CpG dinucleotides along the *UBXN8* promoter region (–1500 to –1 bp, TSS up 1.5 kb). The horizontal line (F-R) indicates the location of the primers used in the BSP assays. **c** A genomic BSP assay was performed to profile the methylation pattern of the CpG dinucleotides located along the RUNX1-binding site. Black circles and empty circles denote methylated and unmethylated CpG dinucleotides, respectively. **d** The mRNA and protein expression of UBXN8 was determined in cells treated with 1.0 µM DAC. Error bars indicate the SD from three different assays.

To further understand the mechanism of *UBXN8* downregulation in RUNX1-RUNX1T1^+^ cell lines, we used the MCC-Seq technique to detect the genome-wide DNA methylation status of RUNX1-RUNX1T1^+/−^ cell lines (SKNO-1 and SKNO-siAE cell lines) and the altered DNA methylation status in cells treated with 5-aza-2 deoxycytidine, and the DNA methylation levels changed in a large number of genes (Fig. 2). To our surprise, we found that the promoter region (–1500 to –1 bp, TSS up 1.5k) of the *UBXN8* gene was significantly hypermethylated in SKNO-1 cells in contrast with SKNO-siAE cells (Fig. 3a). The details of the methylation levels at CpG sites (TSS up 1.5k) for *UBXN8* and the overall sequencing statistics of each sample are shown in additional files 2–3, and the genes with different methylation levels between SKNO-1 and SKNO-siAE are shown in additional files 4–5. We also performed a bioinformatics search (http://www.urogene.org/cgi-bin/methprimer/methprimer.cgi) to analyze the methylation pattern of the *UBXN8* gene promoter region (–1500 to –1 bp, TSS up 1.5k) and identified four CpG islands (criteria used: island size > 100, GC percent > 50.0, Obs/Exp > 0.6) along the promoter region (Fig. 3b). Bisulfite sequencing (BSP) of the *UBXN8* genome promoter region from -967 to -769 bp (primers are indicated in Fig. 3b) revealed that this CpG island was hypermethylated in RUNX1-RUNX1T1^+^ cell lines (SKNO-1 and U-937AE) compared with RUNX1-RUNX1T1^-^ cell lines (SKNO-siAE and U-937) and NBM (Fig. 3c). To further elucidate whether DNA methylation was responsible for the downregulation of *UBXN8* expression, we treated the RUNX1-RUNX1T1^+^ cell lines with 5-aza-2 deoxycytidine (DAC), a DNMT inhibitor, at 1.0 μM for 72 h and found that the mRNA and protein expression levels of UBXN8 were significantly increased (Fig. 3d). Together, these results indicated that in RUNX1-RUNX1T1^+^ AML cell lines, DNA methylation of the *UBXN8* promoter region may contribute to its transcriptional silencing.

### The RUNX1-RUNX1T1 oncoprotein localizes at the RUNX1-binding site on the *UBXN8* genomic promoter region and affects its transcriptional regulation

**Fig. 4:**
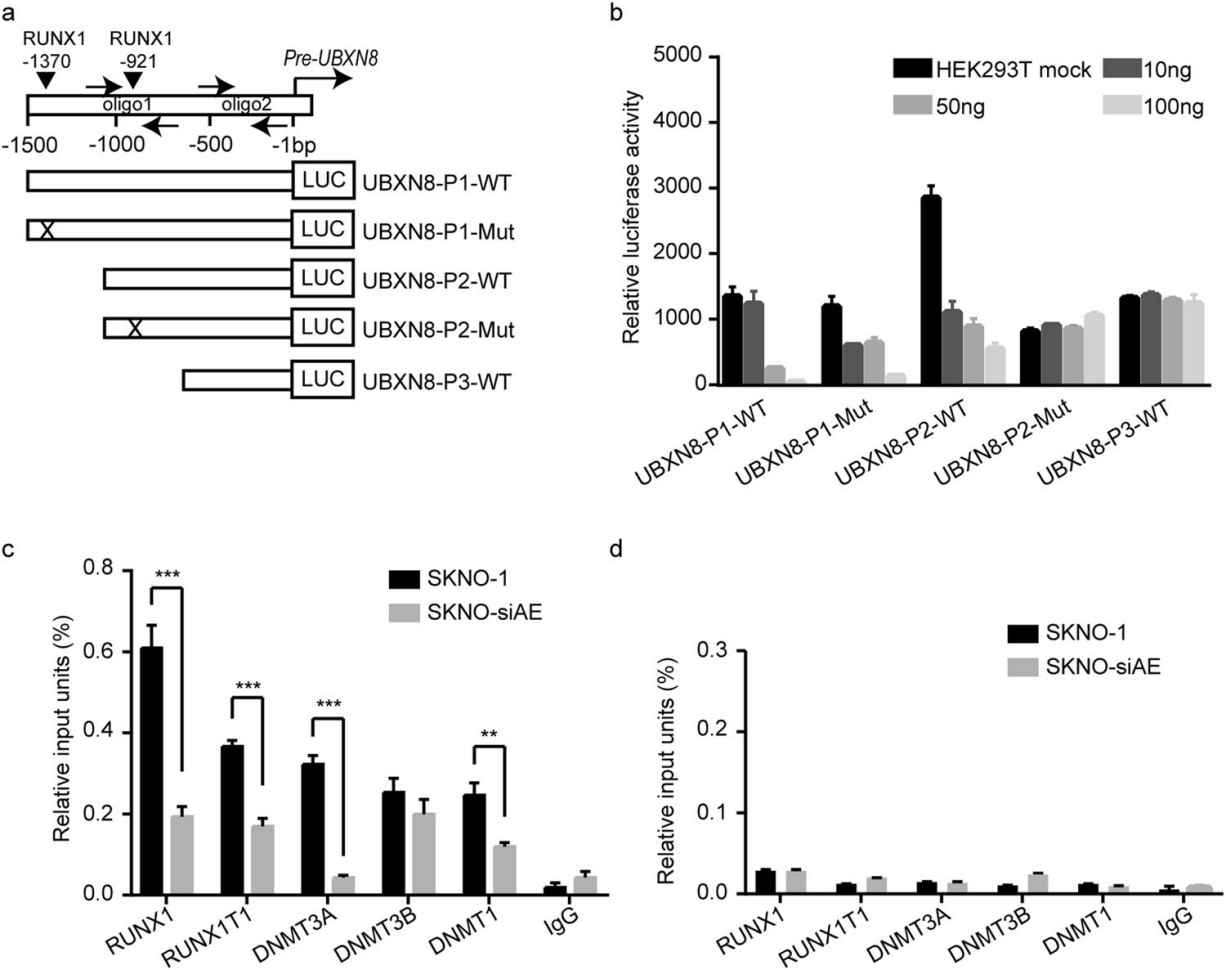
The RUNX1-RUNX1T1 fusion protein acts on the RUNX1-binding site in the *UBXN8* promoter region and triggers the epigenetic silencing of *UBXN8*. **a** Schematic of the RUNX1-binding sites (nt –1370 to –1360, nt –921 to –911) along the *UBXN8* gene promoter region. The numbers refer to the nucleotide positions relative to the 5′ end of the TSS of *UBXN8* ( + 1). Arrows indicate the location of the primers used in the ChIP assay. **b** Human 293T cells were transiently cotransfected with luciferase reporter vectors pGL3-LUC containing the sequence of the regulatory regions (shown in Fig. 4a) and increasing amounts of pcDNA3.0 vectors with or without the *RUNX1-RUNX1T1*-binding sites. Dual-luciferase reporter assays were performed to detect the transcriptional activity of RUNX1-RUNX1T1 on the *UBXN8* promoter region. **c**, **d** Chromatin was immunoprecipitated using the indicated antibodies or IgG. qRT-PCR analysis was performed to amplify the region in *UBXN8* containing the predicted RUNX1-binding site with oligo 1 primers and the distal region lacking the RUNX1 site with oligo 2 primers (shown in Fig. 4a). Amplification of GAPDH was used as a control. Error bars indicate the SD from three different assays (^**^P < 0.01, ^***^P < 0.001).

To address the question of whether the RUNX1-RUNX1T1 oncoprotein mediates *UBXN8* epigenetic silencing in t(8;21) AML, we searched the JASPAR database for RUNX1-binding sites (http://jaspar.binf.ku.dk/cgi-bin/jaspar_db.pl) and found two putative RUNX1-binding sites (–1370 to –1360 bp, –921 to –911 bp) at the 5′ end of the predicted “core-promoter” sequence on the *UBXN8* promoter region (–1500 to –1 bp) (Fig. 4a). To elucidate the transcriptional regulatory effect of the RUNX1-RUNX1T1 oncoprotein on the expression of *UBXN8*, we cloned different lengths of the *UBXN8* promoter sequence with different wild-type (WT) and mutant (Mut) RUNX1-binding sites (Fig. 4a) into a pGL3.0-basic vector (400 ng) and evaluated luciferase activity in 293T cells cotransfected with increasing amounts (10, 50, and 100 ng) of plasmids encoding the RUNX1-RUNX1T1 product or with empty vector (mock). The detected luciferase activity showed that ectopic RUNX1-RUNX1T1 expression resulted in a decrease in luciferase activity in cells expressing UBXN8-P1-WT, UBXN8-P1-Mut and UBXN8-P2-WT, indicating that RUNX1-RUNX1T1 can block the regulatory function of RUNX1 by competing with it at its binding sites (Fig. 4b). By comparing the luciferase activity between UBXN8-P1-WT and UBXN8-P2-WT in cells transfected with empty vector in Fig. 4b, it is obvious that the luciferase activity had a robust decrease in cells transfected with UBXN8-P2-WT and 10 ng of RUNX1-RUNX1T1 compared to cells transfected with UBXN8-P2-WT and empty vector (HEK293T mock).

Therefore, it is clear that RUNX1-RUNX1T1 blocks the regulatory function of RUNX1 by competing with it at its binding sites, and the displacement of RUNX1 with RUNX1-RUNX1T1 at P2 is crucial for the downregulation of *UBXN8* in t(8;21) AML cells. We further performed a ChIP assay in SKNO-1 and SKNO-siAE cells to determine whether RUNX1-RUNX1T1 binds directly to the promoter region and leads to the methylation at the *UBXN8* promoter using anti-RUNX1, anti-RUNX1T1, anti-DNMT3A, anti-DNMT3B, and anti-DNMT1-specific antibodies or a nonspecific antibody (normal IgG). Quantitative RT-PCR was performed with primers specific to the *UBXN8* chromatin regulatory regions surrounding RUNX1-binding site 2 (oligo1 in Fig. 4a) or with unrelated negative control primers (oligo2 in Fig. 4a). The results indicated that RUNX1, RUNX1T1, DNMT3A and DNMT1 were present at the *UBXN8* promoter region surrounding the functional RUNX1-binding site and that the RUNX1-RUNX1T1 oncoprotein together with DNMT3A and DNMT1 might form a complex that methylates the *UBXN8* promoter region (Fig. 4c, d).

### Overexpression of *UBXN8* induces cell cycle arrest and differentiation and reduces proliferation in t(8;21) AML cell lines

**Fig. 5:**
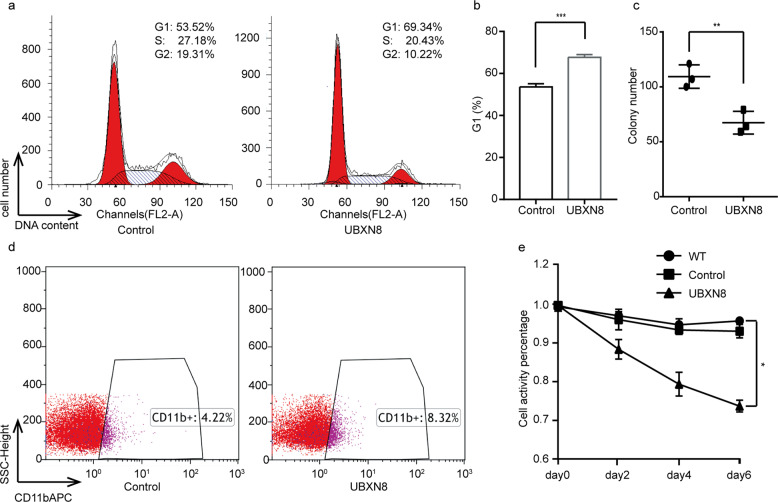
Ectopic *UBXN8* expression induces cell cycle arrest, reprograms myeloid differentiation, suppresses colony formation and inhibits proliferation. **a**, **b** Flow cytometric analysis of cell cycle distribution showed that ectopic *UBXN8* expression induced G1 arrest. **c** A colony formation assay was performed on SKNO-1 cells transduced with control or *UBXN8* lentiviral vector and cultured for 8 days. **d** Flow cytometric analyses of CD11b expression showed that ectopic *UBXN8* expression increased myeloid differentiation. **e** The CCK-8 assay was performed on SKNO-1 cells transduced with control or *UBXN8* lentiviral vector at the indicated time points. Error bars show the SD from three different assays (^*^P < 0.05, ^**^P < 0.01, ^***^P < 0.001).

To evaluate the effects of the *UBXN8* gene on t(8;21) AML, we constructed and selected puromycin-resistant SKNO-1 cells transduced with lenti-UBXN8 or control lentiviral vectors. Cell cycle analysis via flow cytometry of SKNO-1 cells transduced with UBXN8 or control lentiviral vectors revealed that ectopic expression of *UBXN8* significantly increased the fraction of SKNO-1 cells in G1 phase (Fig. 5a, b). In addition, ectopic *UBXN8* expression reprogrammed myeloid differentiation with an increase in the percentage of CD11b from 4.22 to 8.32%, indicating that the restoration of *UBXN8* expression could promote the differentiation of malignant cells (Fig. 5d). The colony formation assay (Fig. 5c) and CCK-8 assay (Fig. 5e) results revealed that restoration of *UBXN8* expression significantly decreased the proliferation ability of SKNO-1 cells.

### *UBXN8* plays a tumor suppressor role in t(8;21) AML in vivo

**Fig. 6:**
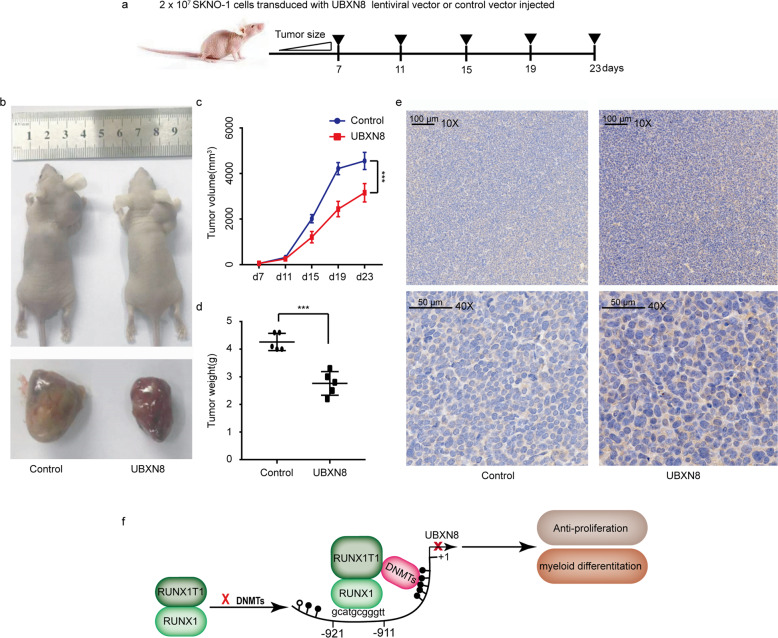
*UBXN8* inhibits leukemic growth and reprograms myeloid differentiation in vivo. **a** Schematic of the experimental design of the xenograft mouse model. **b** Images of two mice and their excised tumors from the UBXN8 and control groups at the end of the experiment. **c** Tumor volumes of the two groups (Control: *n* = 5, UBXN8: *n* = 5) were measured at the indicated days during the experiment. **d** The weights of the tumors from the two groups (Control: *n* = 5, UBXN8: *n* = 5) were measured at the end of the experiment. **e** Ectopic expression of *UBXN8* significantly increased the expression of CD11b in vivo as examined by immunohistochemical analysis (upper panel: scale bar 100 μm, 10X; lower panel: scale bar 50 μm, 40X). **f** Schematic model for the epigenetic silencing of the *UBXN8* gene by RUNX1-RUNX1T1. In myeloid precursor cells, the occupancy of the RUNX1-binding site (GCATGCGGGTT) on the *UBXN8* promoter region by RUNX1 is associated with a chromatin status permissive for transcription. The RUNX1-RUNX1T1 oncoprotein targets this binding site, where it aberrantly recruits DNMTs and methylates CpGs. This induces chromatin packaging that is nonpermissive for *UBXN8* gene transcription, which leads to the inhibition of the differentiation of RUNX1-RUNX1T1^+^ myeloid precursors and consequently promotes myeloid proliferation. Black and white circles indicate methylated and unmethylated CpG dinucleotides, respectively. The numbers indicate the position of the nucleotides relative to the TSS of *UBXN8* ( + 1).

Having demonstrated the relevance of the RUNX1-RUNX1T1 oncoprotein in the regulation of *UBXN8* gene expression and the biological effects of *UBXN8* restoration on SKNO-1 cells in vitro, we focused on evaluating whether these findings could provide a potential viable therapeutic target for RUNX1-RUNX1T1^+^ leukemia in vivo. To this end, we subcutaneously injected approximately 2 × 10^7^ SKNO-1 cells transduced with *UBXN8*-overexpressing or control lentiviral vector into the right flank of immunocompromised nude mice (Fig. 6a). We then measured the largest diameter and the perpendicular diameter of each implanted tumor on the indicated days and photographed the mice in the *UBXN8* and control groups at the end of the experiment (day 23) (Fig. 6b). The average tumor volume of each group was calculated, and the results revealed that the tumor volumes in each group exhibited linear growth, with final average volumes of 4551.8 mm^3^ and 3156.2 mm^3^ in the control and *UBXN8* groups, respectively (*P* < 0.0001) (Fig. 6c). The average tumor weights of the control and *UBXN8* groups were 4.26 g and 2.76 g, respectively (*P* < 0.0001) (Fig. 6d). In addition, CD11b expression was significantly increased in the *UBXN8* group compared to the control group (Fig. 6e). It was obvious that ectopic expression of *UBXN8* significantly inhibited the growth and promoted the differentiation of SKNO-1 cells in vivo. Together, these results indicated that restoration of *UBXN8* expression significantly contributes to the inhibition of malignant cell proliferation and the promotion of malignant cell differentiation, which makes this a promising therapeutic approach for treating t(8;21) AML.

## Discussion

RUNX1-RUNX1T1^+^ leukemia is one of the most common cytogenetic subtypes of AML, and the RUNX1-RUNX1T1 fusion protein plays an important triggering role in the pathogenesis of t(8;21) AML by regulating gene expression through various mechanisms^1,19,20^. Indeed, aberrant DNA methylation is a hallmark of cancer and plays critical roles in the initiation, progression and prognosis of AML^21^. Our previous results showed that RUNX1-RUNX1T1 can bind to the promoter of *miR-193a* and *PTEN* and provided evidence that links the epigenetic silencing of the tumor suppressor genes *miR-193a* and *PTEN* to blocking the differentiation of myeloid precursors^14^. However, the identification of DNA methylation patterns has to date not resulted in clinical applications due to the limitations of detecting DNA methylation, such as high cost, time consumption and difficulty in data processing. Currently, a large number of DNA methylation detection techniques have emerged, and existing techniques are continuously evolving. Among these techniques, MethylC-capture sequencing (MCC-Seq)^11^, which is based on the next-generation sequencing (NGS) capture approach, has the ability to detect functional DNA methylomes, with the characteristic function of customization and a high-cost performance ratio. This technique is based on the Roche NimbleGen SeqCap Epi CpGiant enrichment system with a specific design and long probes, which allows for increased accuracy compared to other techniques and more effective cataloging of the functional and disease-related methylation variants for large-scale epigenome-wide methylation studies, especially for heterogeneous diseases such as AML^3^.

To date, there has been little research on the *UBXN8* gene; correspondingly, the exact features and functions of this gene are still unclear. It has been reported that the *UBXN8* gene encodes one of the p97 cofactors, but its role in tumor biology is not yet well characterized. This cofactor is a transmembrane protein that localizes to the endoplasmic reticulum (ER) membrane and can tether p97 to the ER membrane *via* its UBX domain^6^. The association of this cofactor with p97 facilitates the efficient endoplasmic reticulum-associated degradation (ERAD) of misfolded proteins. Insufficient expression of UBXN8 may disturb this process, leading to the accumulation of misfolded proteins in the ER lumen and subsequently inducing the unfolded protein response (UPR) or ER stress, which could induce the degradation of TP53^22^. According to the literature, the ERAD pathway is not only important for maintaining the homeostasis of cells by degrading misfolded proteins but is also critical for the regulation of physiological processes^23^. Thus, it is not surprising that nearly 70 human diseases have been linked to the ERAD pathway^24^. Although the p97 protein has been linked to malignancy^25^, the role of its cofactor UBXN8 is less clear. A recent study reported that the expression of UBXN8 was lower in HCC tissue than in the surrounding normal tissue and suggested that it is a novel tumor suppressor candidate that functions in a TP53-dependent manner^6,10^. However, there are still no relevant reports about the role of UBXN8 in AML. Among the genes with varied methylation in RUNX1-RUNX1T1^+^ leukemia cells, *UBXN8* has obvious hypermethylation at its promoter region, and evidence from several bioinformatic databases shows that *UBXN8* is obviously downregulated in RUNX1-RUNX1T1^+^ leukemia, which garnered our attention.

This study, for the first time, reports that ectopic expression of *UBXN8* can induce G1 arrest, inhibit cell proliferation and induce the differentiation of SKNO-1 cells. In vivo experiments demonstrated that ectopic expression of the *UBXN8* gene can clearly decrease tumor proliferation and promote the expression of CD11b in mice engrafted with SKNO-1 cells, further confirming the in vitro results. However, our study was still limited due to the lack of mechanistic studies of how UBXN8 exerts its growth suppressive functions or induces differentiation.

We also showed for the first time that hypermethylation of the promoter region of the *UBXN8* gene leads to the specific downregulation of UBXN8 expression in RUNX1-RUNX1T1^+^ leukemia and that the RUNX1-RUNX1T1 oncoprotein can bind to the RUNX1-binding site along the *UBXN8* gene promoter sequence; this protein in turn can recruit DNMT3A and DNMT1 to form a complex and methylate CpG islands within the *UBXN8* promoter, thus blocking its transcription (Fig. 6f). In other words, the RUNX1-RUNX1T1 oncoprotein, as the triggering factor, mediates epigenetic inactivation and gene silencing of the *UBXN8* gene in t(8;21) AML, which blocks the specific antitumor effect of the *UBXN8* gene against t(8;21) AML. However, the exact mechanism underlying the antitumor effect of the *UBXN8* gene in t(8;21) AML remains unclear and requires further research.

In conclusion, our results revealed new insight into the role of the *UBXN8* gene in leukemogenesis in t(8;21) AML. We demonstrated that the *UBXN8* gene plays a role as a tumor suppressor in vitro/in vivo and is specifically silenced in t(8;21) AML *via* promoter methylation. We suggest that manipulating *UBXN8* expression is a novel strategy for the treatment of t(8;21) AML.

## Supplementary information

Additional files

Additional files 5

## Data Availability

The MCC-Seq dataset analyzed in this study is available in GEO under accession number GSE155466, while it remains in private status with the access code olqduqyqbhqvtyb for reviewers only.

## Materials availability

We declare that the materials described in the manuscript, including all the relevant raw data, will be available from the corresponding author on reasonable request.
