# Supplementary material for: Epigenetic silencing of UBXN8 contributes to leukemogenesis in t(8;21) acute myeloid leukemia
Source: Exp Mol Med. 2021 Dec 17;53(12):1902–10. doi: 10.1038/s12276-021-00695-8 (PMC8741982; doi:10.1038/s12276-021-00695-8)

**Additional File 1. Table for primers.**

| No. | Name of primer | Sequence of fragments |
| --- | --- | --- |
| 1 | AE-siAGF1-a | 5’-CCUCGAAAUCGUACUGAGAAG-3’ |
|  | AE-siAGF1-b | 5’-UUGGAGCUUAGCAUGACUCU-3’ |
| 2 | GAPDH-RT-F | 5’-GAGTCAACGGATTTGGTCGT-3’ |
|  | GADPH-RT-R | 5’-TTGATTTTGGAGGGATCTCG-3’ |
| 3 | UBXN8-RT-F | 5’-AGGCCAGCAGATACATAGAGAA-3’ |
|  | UBXN8-RT-R | 5’-GGCTTCACCCGTCATTTGATAAA-3’ |
| 4 | UBXN8-BSP-F | 5’-TTTAGGTTGGAGTGTAGTGGTGTAA-3’ |
|  | UBXN8-BSP-R | 5’-AATCACAAAATCAAAAATTCCAAAC-3’ |
| 5 | UBXN8-ChIP-F1 | 5’-AGTGTTGGGATTACAGGCGTGAG-3’ |
|  | UBXN8-ChIP-R1 | 5’-GGAGGTTGCTTTGAGCCGAGTTT-3’ |
| 6 | UBXN8-ChIP-F2 | 5’-CCGTTAGGGAGATAAGGAAGGTGTT-3’ |
|  | UBXN8-ChIP-R2 | 5’-CCGTGTTTCAGAAGAAAGGCAAGT-3’ |

**Additional File 2.**

| **Chr** | **CpG position** | **Methylation** | | **Promoter region** | | **Gene symbol** | **Sequencing depth** | |
| --- | --- | --- | --- | --- | --- | --- | --- | --- |
|  |  | **Skno-1** | **Skno-siAE** | **start** | **end** |  | **Skno-1** | **Skno-siAE** |
| chr8 | 30601101 | 1 | 1 | 30600181 | 30601680 | UBXN8 | 5 | 17 |
| chr8 | 30601103 | 1 | 0.88888889 | 30600181 | 30601680 | UBXN8 | 5 | 18 |
| chr8 | 30601127 | 1 | 1 | 30600181 | 30601680 | UBXN8 | 5 | 17 |
| chr8 | 30601142 | 1 | 0.29166667 | 30600181 | 30601680 | UBXN8 | 9 | 24 |
| chr8 | 30601168 | 0 | 0.17241379 | 30600181 | 30601680 | UBXN8 | 9 | 29 |
| chr8 | 30601172 | 0 | 0.44827586 | 30600181 | 30601680 | UBXN8 | 9 | 29 |
| chr8 | 30601211 | 0.66666667 | 0 | 30600181 | 30601680 | UBXN8 | 12 | 28 |
| chr8 | 30601224 | 1 | 0.03571429 | 30600181 | 30601680 | UBXN8 | 12 | 28 |
| chr8 | 30601242 | 0.66666667 | 0.03571429 | 30600181 | 30601680 | UBXN8 | 12 | 28 |
| chr8 | 30601246 | 0.66666667 | 0 | 30600181 | 30601680 | UBXN8 | 12 | 28 |
| chr8 | 30601266 | 0.66666667 | 0 | 30600181 | 30601680 | UBXN8 | 12 | 20 |
| chr8 | 30601311 | 0.33333333 | 0 | 30600181 | 30601680 | UBXN8 | 9 | 21 |
| chr8 | 30601323 | 0 | 0 | 30600181 | 30601680 | UBXN8 | 9 | 21 |
| chr8 | 30601365 | 0.4 | 0 | 30600181 | 30601680 | UBXN8 | 20 | 18 |
| chr8 | 30601387 | 0.59259259 | 0 | 30600181 | 30601680 | UBXN8 | 27 | 6 |
| chr8 | 30601388 | 0.30434783 | 0 | 30600181 | 30601680 | UBXN8 | 23 | 34 |
| chr8 | 30601401 | 0.52173913 | 0 | 30600181 | 30601680 | UBXN8 | 23 | 19 |
| chr8 | 30601402 | 0.51428571 | 0 | 30600181 | 30601680 | UBXN8 | 35 | 31 |
| chr8 | 30601413 | 0.43333333 | 0 | 30600181 | 30601680 | UBXN8 | 30 | 19 |
| chr8 | 30601414 | 0.5625 | 0 | 30600181 | 30601680 | UBXN8 | 32 | 32 |
| chr8 | 30601454 | 0.51612903 | 0 | 30600181 | 30601680 | UBXN8 | 31 | 21 |
| chr8 | 30601455 | 0.47826087 | 0 | 30600181 | 30601680 | UBXN8 | 46 | 48 |
| chr8 | 30601499 | 0 | 0 | 30600181 | 30601680 | UBXN8 | 26 | 27 |
| chr8 | 30601500 | 0.21621622 | 0 | 30600181 | 30601680 | UBXN8 | 37 | 34 |
| chr8 | 30601522 | 0.33333333 | 0 | 30600181 | 30601680 | UBXN8 | 12 | 28 |
| chr8 | 30601523 | 0.19444444 | 0 | 30600181 | 30601680 | UBXN8 | 36 | 28 |
| chr8 | 30601548 | 0 | 0 | 30600181 | 30601680 | UBXN8 | 15 | 27 |
| chr8 | 30601549 | 0.16981132 | 0 | 30600181 | 30601680 | UBXN8 | 53 | 29 |
| chr8 | 30601572 | 0.35 | 0 | 30600181 | 30601680 | UBXN8 | 20 | 28 |
| chr8 | 30601573 | 0.48780488 | 0 | 30600181 | 30601680 | UBXN8 | 41 | 10 |
| chr8 | 30601577 | 0.3 | 0 | 30600181 | 30601680 | UBXN8 | 20 | 26 |
| chr8 | 30601578 | 0.33333333 | 0 | 30600181 | 30601680 | UBXN8 | 30 | 10 |
| chr8 | 30601581 | 0.35 | 0 | 30600181 | 30601680 | UBXN8 | 20 | 26 |
| chr8 | 30601582 | 0.43333333 | 0 | 30600181 | 30601680 | UBXN8 | 30 | 10 |
| chr8 | 30601596 | 0.31578947 | 0 | 30600181 | 30601680 | UBXN8 | 19 | 26 |
| chr8 | 30601597 | 0.39393939 | 0 | 30600181 | 30601680 | UBXN8 | 33 | 13 |
| chr8 | 30601617 | 0 | 0 | 30600181 | 30601680 | UBXN8 | 7 | 24 |
| chr8 | 30601618 | 0 | 0 | 30600181 | 30601680 | UBXN8 | 30 | 12 |
| chr8 | 30601622 | 0.68181818 | 0 | 30600181 | 30601680 | UBXN8 | 22 | 23 |
| chr8 | 30601623 | 0.48148148 | 0 | 30600181 | 30601680 | UBXN8 | 27 | 13 |
| chr8 | 30601624 | 0.68181818 | 0.19047619 | 30600181 | 30601680 | UBXN8 | 22 | 21 |
| chr8 | 30601625 | 0.48148148 | 0 | 30600181 | 30601680 | UBXN8 | 27 | 13 |
| chr8 | 30601650 | 0.6 | 0 | 30600181 | 30601680 | UBXN8 | 35 | 6 |
| chr8 | 30601651 | 0.58823529 | 0 | 30600181 | 30601680 | UBXN8 | 34 | 17 |
| chr8 | 30601658 | 0.58823529 | 0 | 30600181 | 30601680 | UBXN8 | 34 | 11 |
| chr8 | 30601660 | 0.58823529 | 0 | 30600181 | 30601680 | UBXN8 | 34 | 11 |
| chr8 | 30601663 | 0.6 | 0 | 30600181 | 30601680 | UBXN8 | 35 | 7 |
| chr8 | 30601664 | 0.55172414 | 0 | 30600181 | 30601680 | UBXN8 | 29 | 11 |
| chr8 | 30601672 | 0.61111111 | 0 | 30600181 | 30601680 | UBXN8 | 36 | 11 |
| chr8 | 30601673 | 0.5862069 | 0 | 30600181 | 30601680 | UBXN8 | 29 | 12 |

**Additional File 3.**

|  | | SKNO-1 | SKNO-siAE |
| --- | --- | --- | --- |
| **Raw data** | **Raw_Reads** | 41729572.00 | 40210216.00 |
|  | **Raw_base** | 5216196500.00 | 5026277000.00 |
| **Clean data** | **Clean_Reads** | 41676054.00 | 40160696.00 |
|  | **Clean_base** | 5197976147.00 | 5008818065.00 |
|  | **Clean_rate** | 1.00 | 1.00 |
| **Map Reads** | **paired_map** | 19248466.00 | 18559246.00 |
|  | **singleA_map** | 1101196.00 | 1060675.00 |
|  | **singleB_map** | 675080.00 | 654611.00 |
|  | **Map_rate** | 0.97 | 0.97 |
| **Depth-CG** | | 12.57 | 12.58 |
| **Genome CpG sites** | | 56434896.00 | 56434896.00 |
| **Coverage CpG sites** | **1X** | 21000398.00 | 21797882.00 |
|  | **rate-1X** | 0.37 | 0.39 |
|  | **5X** | 4713346.00 | 4694016.00 |
|  | **rate-5X** | 0.08 | 0.08 |
|  | **10X** | 2858983.00 | 2844971.00 |
|  | **rate-10X** | 0.05 | 0.05 |
|  | **20X** | 1349076.00 | 1325531.00 |
|  | **rate-20X** | 0.02 | 0.02 |
| **Methylation (5X.CG)** | | 0.58 | 0.65 |
| **Conversion rate (%)** | | 99.47 | 99.55 |

**Additional File 4.**

| **DMR** | | | **q-value** | **D-value** | **#CpG** | **p-value**  **(MWU-test)** | **p-value**  **(2D KS-test)** | **Methylated level** | | **Gene Symbol** |
| --- | --- | --- | --- | --- | --- | --- | --- | --- | --- | --- |
| **Chr** | **start** | **end** |  |  |  |  |  | **SKNO-1** | **SKNO-siAE** |  |
| chr9 | 99540231 | 99540677 | 3.40E-12 | 0.9896 | 53 | 4.60E-14 | 2.90E-18 | 0.9923 | 0.0027 | ZNF510 |
| chr4 | 1.77E+08 | 1.77E+08 | 0.00018 | 0.9827 | 32 | 6.30E-12 | 1.50E-10 | 0.9864 | 0.0037 | WDR17 |
| chr1 | 12123556 | 12123725 | 0.0014 | 0.9688 | 26 | 6.20E-10 | 1.20E-09 | 0.9927 | 0.0239 | TNFRSF8 |
| chr4 | 1.59E+08 | 1.59E+08 | 3.10E-09 | 0.9684 | 52 | 3.90E-14 | 2.60E-15 | 0.9762 | 0.0078 | TMEM144 |
| chr3 | 1.57E+08 | 1.57E+08 | 2.00E-10 | 0.9668 | 47 | 4.70E-14 | 1.70E-16 | 0.9708 | 0.0039 | LINC00886 |
| chr19 | 58399819 | 58400625 | 3.00E-10 | 0.9659 | 54 | 4.00E-14 | 2.60E-16 | 0.9783 | 0.0125 | ZNF814 |
| chr5 | 1.26E+08 | 1.26E+08 | 3.60E-19 | 0.9616 | 81 | 4.40E-14 | 3.10E-25 | 0.9716 | 0.0100 | C5orf63 |
| chr5 | 17216882 | 17217035 | 2.00E-07 | 0.9613 | 39 | 7.30E-14 | 1.70E-13 | 0.9718 | 0.0106 | BASP1 |
| chr3 | 1.73E+08 | 1.73E+08 | 2.40E-13 | 0.9593 | 67 | 4.10E-14 | 2.10E-19 | 0.9637 | 0.0045 | NLGN1 |
| chr17 | 5094933 | 5095261 | 8.10E-07 | 0.9588 | 37 | 1.80E-13 | 7.00E-13 | 0.9678 | 0.0090 | LOC100130950 |
| chr17 | 5094933 | 5095261 | 8.10E-07 | 0.9588 | 37 | 1.80E-13 | 7.00E-13 | 0.9678 | 0.0090 | ZNF594 |
| chr18 | 76828369 | 76828738 | 0.0012 | 0.9577 | 26 | 2.70E-09 | 1.10E-09 | 0.9962 | 0.0385 | ATP9B |
| chr5 | 60457646 | 60458421 | 1.50E-05 | 0.9540 | 33 | 3.20E-12 | 1.30E-11 | 0.9742 | 0.0202 | CTC-436P18.1 |
| chr5 | 60457646 | 60458421 | 1.50E-05 | 0.9540 | 33 | 3.20E-12 | 1.30E-11 | 0.9742 | 0.0202 | SMIM15 |
| chr1 | 2.45E+08 | 2.45E+08 | 5.70E-05 | 0.9525 | 29 | 6.20E-11 | 4.90E-11 | 0.9594 | 0.0069 | C1orf101 |
| chr19 | 37406805 | 37407890 | 4.70E-28 | 0.9523 | 107 | 4.60E-14 | 4.10E-34 | 0.9561 | 0.0038 | ZNF568 |
| chr19 | 37406805 | 37407890 | 4.70E-28 | 0.9523 | 107 | 4.60E-14 | 4.10E-34 | 0.9561 | 0.0038 | ZNF829 |
| chr7 | 149724 | 150116 | 1.20E-09 | 0.9515 | 47 | 4.10E-14 | 1.10E-15 | 0.9594 | 0.0080 | LOC100507642 |
| chr7 | 149724 | 150116 | 1.20E-09 | 0.9515 | 47 | 4.10E-14 | 1.10E-15 | 0.9594 | 0.0080 | LOC102723672 |
| chr21 | 37442103 | 37442568 | 4.90E-20 | 0.9508 | 79 | 4.40E-14 | 4.20E-26 | 0.9528 | 0.0020 | CBR1 |
| chr1 | 1.61E+08 | 1.61E+08 | 8.90E-10 | 0.9501 | 46 | 4.40E-14 | 7.60E-16 | 0.9566 | 0.0065 | NDUFS2 |
| chr3 | 1.85E+08 | 1.85E+08 | 1.10E-16 | 0.9495 | 68 | 4.00E-14 | 9.80E-23 | 0.9505 | 0.0010 | MAP3K13 |
| chr6 | 1.63E+08 | 1.63E+08 | 1.30E-10 | 0.9484 | 50 | 4.10E-14 | 1.10E-16 | 0.9580 | 0.0096 | PACRG |
| chr6 | 1.63E+08 | 1.63E+08 | 1.30E-10 | 0.9484 | 50 | 4.10E-14 | 1.10E-16 | 0.9580 | 0.0096 | PARK2 |
| chr19 | 12250695 | 12251601 | 8.30E-12 | 0.9480 | 56 | 4.70E-14 | 7.10E-18 | 0.9664 | 0.0184 | ZNF20 |
| chr13 | 80055180 | 80056117 | 1.70E-19 | 0.9477 | 85 | 4.20E-14 | 1.50E-25 | 0.9792 | 0.0315 | NDFIP2-AS1 |
| chr13 | 80055180 | 80056117 | 1.70E-19 | 0.9477 | 85 | 4.20E-14 | 1.50E-25 | 0.9792 | 0.0315 | NDFIP2 |
| chr19 | 19931955 | 19932791 | 2.10E-14 | 0.9430 | 64 | 4.30E-14 | 1.80E-20 | 0.9543 | 0.0113 | ZNF506 |
| chr5 | 1.59E+08 | 1.59E+08 | 1.50E-21 | 0.9419 | 90 | 4.80E-14 | 1.30E-27 | 0.9519 | 0.0100 | UBLCP1 |
| chr9 | 38620463 | 38621070 | 0.00092 | 0.9355 | 43 | 1.30E-09 | 7.90E-10 | 0.9393 | 0.0233 | ANKRD18A |
| chr9 | 38620463 | 38621070 | 0.00092 | 0.9355 | 43 | 1.30E-09 | 7.90E-10 | 0.9393 | 0.0233 | FAM201A |
| chr13 | 96705545 | 96705930 | 5.50E-08 | 0.9330 | 45 | 4.20E-14 | 4.80E-14 | 0.9377 | 0.0047 | UGGT2 |
| chr5 | 17217853 | 17219239 | 4.10E-33 | 0.9316 | 127 | 4.60E-14 | 3.50E-39 | 0.9361 | 0.0044 | LOC285696 |
| chr19 | 56988268 | 56988983 | 6.30E-11 | 0.9303 | 53 | 4.60E-14 | 5.40E-17 | 0.9325 | 0.0022 | ZNF667-AS1 |
| chr19 | 56988268 | 56988983 | 6.30E-11 | 0.9303 | 53 | 4.60E-14 | 5.40E-17 | 0.9325 | 0.0022 | ZNF667 |
| chr9 | 38619727 | 38620371 | 1.10E-09 | 0.9283 | 51 | 4.40E-14 | 9.40E-16 | 0.9381 | 0.0098 | ANKRD18A |
| chr9 | 38619727 | 38620371 | 1.10E-09 | 0.9283 | 51 | 4.40E-14 | 9.40E-16 | 0.9381 | 0.0098 | FAM201A |
| chr19 | 37701240 | 37701739 | 5.30E-10 | 0.9275 | 49 | 4.30E-14 | 4.60E-16 | 0.9567 | 0.0292 | ZNF585B |
| chr20 | 49548314 | 49548525 | 0.00077 | 0.9273 | 31 | 1.30E-11 | 6.70E-10 | 0.9614 | 0.0341 | ADNP |
| chr19 | 31841625 | 31842291 | 0.0033 | 0.9264 | 29 | 6.20E-11 | 2.90E-09 | 0.9439 | 0.0175 | TSHZ3 |
| chr7 | 82072916 | 82073300 | 1.30E-11 | 0.9239 | 50 | 4.30E-14 | 1.10E-17 | 0.9327 | 0.0089 | CACNA2D1 |
| chr21 | 28339843 | 28340089 | 0.0024 | 0.9218 | 24 | 2.90E-09 | 2.10E-09 | 0.9462 | 0.0244 | ADAMTS5 |
| chr1 | 2.03E+08 | 2.03E+08 | 0.00035 | 0.9217 | 30 | 2.90E-11 | 3.00E-10 | 0.9579 | 0.0362 | PPFIA4 |
| chr8 | 1.45E+08 | 1.45E+08 | 1.40E-05 | 0.9200 | 33 | 3.20E-12 | 1.20E-11 | 0.9414 | 0.0214 | MROH6 |
| chr7 | 28220448 | 28220690 | 0.00013 | 0.9200 | 29 | 6.20E-11 | 1.10E-10 | 0.9678 | 0.0479 | JAZF1-AS1 |
| chr7 | 28220448 | 28220690 | 0.00013 | 0.9200 | 29 | 6.20E-11 | 1.10E-10 | 0.9678 | 0.0479 | JAZF1 |
| chr1 | 84465045 | 84465500 | 7.50E-06 | 0.9173 | 35 | 7.30E-13 | 6.50E-12 | 0.9675 | 0.0501 | TTLL7 |
| chr8 | 79577781 | 79578206 | 8.50E-05 | 0.9167 | 33 | 2.90E-12 | 7.30E-11 | 0.9181 | 0.0014 | ZC2HC1A |
| chr6 | 84140586 | 84140997 | 3.10E-05 | 0.9136 | 31 | 1.30E-11 | 2.70E-11 | 0.9261 | 0.0124 | ME1 |
| chr3 | 1.98E+08 | 1.98E+08 | 0.0018 | 0.9120 | 36 | 3.40E-13 | 1.50E-09 | 0.9308 | 0.0188 | ANKRD18DP |
| chr5 | 1.1E+08 | 1.1E+08 | 9.80E-08 | 0.9068 | 43 | 4.70E-14 | 8.40E-14 | 0.9654 | 0.0587 | TSLP |
| chr19 | 37825445 | 37826018 | 5.40E-08 | 0.9048 | 40 | 5.70E-14 | 4.60E-14 | 0.9691 | 0.0643 | HKR1 |
| chr19 | 19495908 | 19496138 | 0.00037 | 0.9040 | 31 | 1.50E-11 | 3.20E-10 | 0.9497 | 0.0458 | GATAD2A |
| chr7 | 76026756 | 76027138 | 3.50E-06 | 0.9035 | 35 | 3.50E-12 | 3.00E-12 | 0.9235 | 0.0200 | ZP3 |
| chr7 | 915753 | 915872 | 3.50E-06 | 0.9028 | 36 | 3.40E-13 | 3.00E-12 | 0.9068 | 0.0040 | GET4 |
| chr19 | 33794324 | 33794840 | 5.10E-09 | 0.9023 | 41 | 4.90E-14 | 4.30E-15 | 0.9536 | 0.0513 | CEBPA |
| chr17 | 47075067 | 47075351 | 2.30E-07 | 0.9019 | 43 | 4.70E-14 | 2.00E-13 | 0.9032 | 0.0013 | IGF2BP1 |
| chr19 | 7991718 | 7992086 | 0.00013 | 0.8999 | 29 | 6.20E-11 | 1.10E-10 | 0.9284 | 0.0286 | CTXN1 |
| chr11 | 65600739 | 65600911 | 0.0077 | 0.8996 | 27 | 2.90E-10 | 6.60E-09 | 0.9104 | 0.0108 | SNX32 |
| chr6 | 6004349 | 6005431 | 2.60E-19 | 0.8994 | 84 | 4.80E-14 | 2.20E-25 | 0.9207 | 0.0213 | NRN1 |
| chr7 | 27224162 | 27224362 | 8.00E-08 | 0.8992 | 42 | 4.70E-14 | 6.90E-14 | 0.9385 | 0.0393 | HOXA11-AS |
| chr1 | 1.51E+08 | 1.51E+08 | 0.003 | 0.8944 | 28 | 1.30E-10 | 2.60E-09 | 0.9515 | 0.0571 | GOLPH3L |
| chr17 | 61523198 | 61523529 | 1.80E-08 | 0.8933 | 42 | 4.80E-14 | 1.50E-14 | 0.9500 | 0.0567 | CYB561 |
| chr10 | 1.35E+08 | 1.35E+08 | 8.30E-18 | 0.8898 | 79 | 4.50E-14 | 7.10E-24 | 0.9413 | 0.0515 | TUBGCP2 |
| chr3 | 1.26E+08 | 1.26E+08 | 8.90E-05 | 0.8880 | 36 | 3.90E-13 | 7.60E-11 | 0.9269 | 0.0389 | KLF15 |
| chr7 | 1.03E+08 | 1.03E+08 | 1.70E-05 | 0.8878 | 35 | 6.70E-13 | 1.50E-11 | 0.8918 | 0.0040 | NAPEPLD |
| chr11 | 32455013 | 32455639 | 1.20E-08 | 0.8874 | 49 | 4.40E-14 | 1.00E-14 | 0.9650 | 0.0775 | WT1-AS |
| chr18 | 53257169 | 53257417 | 3.40E-07 | 0.8813 | 40 | 5.70E-14 | 3.00E-13 | 0.9063 | 0.0250 | TCF4 |
| chr15 | 1.02E+08 | 1.02E+08 | 1.80E-07 | 0.8812 | 41 | 5.30E-14 | 1.50E-13 | 0.9237 | 0.0425 | TM2D3 |
| chr7 | 1.56E+08 | 1.56E+08 | 1.70E-05 | 0.8804 | 35 | 6.70E-13 | 1.50E-11 | 0.8845 | 0.0041 | C7orf13 |
| chr7 | 1.56E+08 | 1.56E+08 | 1.70E-05 | 0.8804 | 35 | 6.70E-13 | 1.50E-11 | 0.8845 | 0.0041 | RNF32 |
| chr8 | 75233559 | 75234149 | 3.70E-12 | 0.8797 | 56 | 4.70E-14 | 3.20E-18 | 0.8888 | 0.0091 | JPH1 |
| chr21 | 34397862 | 34398533 | 3.10E-05 | 0.8788 | 32 | 6.30E-12 | 2.70E-11 | 0.8798 | 0.0009 | OLIG2 |
| chr1 | 2.21E+08 | 2.21E+08 | 5.20E-12 | 0.8787 | 56 | 4.60E-14 | 4.40E-18 | 0.9597 | 0.0810 | HLX-AS1 |
| chr17 | 79374068 | 79374982 | 1.20E-31 | 0.8743 | 119 | 4.70E-14 | 1.00E-37 | 0.8789 | 0.0046 | MIR4740 |
| chr19 | 37288426 | 37288896 | 4.00E-13 | 0.8734 | 64 | 4.30E-14 | 3.40E-19 | 0.8898 | 0.0163 | ZNF790-AS1 |
| chr4 | 1.29E+08 | 1.29E+08 | 4.00E-06 | 0.8726 | 49 | 4.40E-14 | 3.40E-12 | 0.8864 | 0.0138 | INTU |
| chr19 | 44405669 | 44406074 | 7.60E-06 | 0.8689 | 39 | 7.30E-14 | 6.50E-12 | 0.9420 | 0.0731 | LOC100505715 |
| chr14 | 62217698 | 62218077 | 0.0036 | 0.8677 | 28 | 5.90E-10 | 3.10E-09 | 0.8728 | 0.0051 | HIF1A-AS2 |
| chr5 | 16935845 | 16936035 | 5.30E-10 | 0.8665 | 48 | 4.60E-14 | 4.60E-16 | 0.8740 | 0.0074 | MYO10 |
| chr8 | 1.44E+08 | 1.44E+08 | 0.00016 | 0.8641 | 32 | 6.90E-12 | 1.40E-10 | 0.9022 | 0.0381 | RHPN1-AS1 |
| chr3 | 1.8E+08 | 1.8E+08 | 1.90E-08 | 0.8602 | 43 | 4.70E-14 | 1.70E-14 | 0.8633 | 0.0030 | TTC14 |
| chr19 | 44952298 | 44952867 | 1.10E-08 | 0.8541 | 48 | 4.50E-14 | 9.10E-15 | 0.8618 | 0.0077 | ZNF229 |
| chr1 | 1.52E+08 | 1.52E+08 | 3.10E-07 | 0.8528 | 40 | 6.00E-14 | 2.70E-13 | 0.8917 | 0.0389 | THEM4 |
| chr11 | 63258551 | 63258756 | 5.50E-06 | 0.8520 | 31 | 1.30E-11 | 4.70E-12 | 0.8563 | 0.0043 | HRASLS5 |
| chr22 | 44208082 | 44208202 | 0.00081 | 0.8518 | 31 | 2.70E-10 | 6.90E-10 | 0.8652 | 0.0134 | EFCAB6 |
| chr6 | 16762500 | 16763043 | 0.00072 | 0.8510 | 30 | 2.90E-11 | 6.20E-10 | 0.8644 | 0.0134 | ATXN1 |
| chr1 | 40420210 | 40420606 | 0.0017 | 0.8422 | 32 | 7.40E-11 | 1.50E-09 | 0.9114 | 0.0692 | MFSD2A |
| chr5 | 1.14E+08 | 1.14E+08 | 5.80E-12 | 0.8370 | 55 | 4.00E-14 | 5.00E-18 | 0.9788 | 0.1418 | KCNN2 |
| chr8 | 41754725 | 41754872 | 0.00017 | 0.8355 | 36 | 4.50E-13 | 1.50E-10 | 0.8959 | 0.0605 | ANK1 |
| chr2 | 1.72E+08 | 1.72E+08 | 0.00067 | 0.8293 | 29 | 9.40E-11 | 5.70E-10 | 0.8502 | 0.0209 | GORASP2 |
| chr3 | 1.71E+08 | 1.71E+08 | 2.00E-05 | 0.8257 | 37 | 2.30E-13 | 1.70E-11 | 0.8380 | 0.0123 | TNIK |
| chr10 | 21814277 | 21814455 | 1.30E-05 | 0.8208 | 32 | 6.30E-12 | 1.20E-11 | 0.8328 | 0.0120 | SKIDA1 |
| chr15 | 45670382 | 45671360 | 1.30E-13 | 0.8180 | 72 | 4.00E-14 | 1.20E-19 | 0.8607 | 0.0427 | GATM |
| chr19 | 54024476 | 54024840 | 0.00076 | 0.8146 | 31 | 1.30E-11 | 6.50E-10 | 0.8521 | 0.0375 | ZNF331 |
| chr19 | 49250180 | 49250616 | 0.0022 | 0.8049 | 24 | 2.90E-09 | 1.90E-09 | 0.8276 | 0.0228 | IZUMO1 |
| chr3 | 1.96E+08 | 1.96E+08 | 4.00E-05 | 0.8004 | 36 | 4.90E-13 | 3.40E-11 | 0.8656 | 0.0652 | TM4SF19 |
| chr3 | 1.96E+08 | 1.96E+08 | 4.00E-05 | 0.8004 | 36 | 4.90E-13 | 3.40E-11 | 0.8656 | 0.0652 | TM4SF19-TCTEX1D2 |
| chr19 | 44645378 | 44646069 | 4.00E-20 | 0.7998 | 92 | 3.70E-14 | 3.50E-26 | 0.8295 | 0.0297 | ZNF234 |
| chr19 | 58951522 | 58951896 | 1.30E-08 | 0.7952 | 48 | 4.50E-14 | 1.10E-14 | 0.9464 | 0.1512 | ZNF132 |
| chr6 | 10419399 | 10419492 | 0.0058 | 0.7890 | 24 | 2.90E-09 | 5.00E-09 | 0.9468 | 0.1577 | TFAP2A |
| chr1 | 2.44E+08 | 2.44E+08 | 3.50E-07 | 0.7791 | 38 | 2.40E-13 | 3.00E-13 | 0.9305 | 0.1515 | ZBTB18 |
| chr6 | 1.26E+08 | 1.26E+08 | 8.40E-06 | 0.7765 | 38 | 1.10E-13 | 7.20E-12 | 0.9752 | 0.1987 | HEY2 |
| chr3 | 44903188 | 44903463 | 2.30E-07 | 0.7713 | 37 | 1.80E-13 | 2.00E-13 | 0.7826 | 0.0113 | MIR564 |
| chr3 | 44903188 | 44903463 | 2.30E-07 | 0.7713 | 37 | 1.80E-13 | 2.00E-13 | 0.7826 | 0.0113 | TMEM42 |
| chr19 | 52391010 | 52391481 | 3.10E-14 | 0.7700 | 65 | 4.30E-14 | 2.60E-20 | 0.7790 | 0.0090 | ZNF577 |
| chr19 | 52391010 | 52391481 | 3.10E-14 | 0.7700 | 65 | 4.30E-14 | 2.60E-20 | 0.7790 | 0.0090 | ZNF649-AS1 |
| chr17 | 47072788 | 47074061 | 1.10E-09 | 0.7681 | 60 | 4.30E-14 | 9.80E-16 | 0.8285 | 0.0604 | IGF2BP1 |
| chr6 | 1.5E+08 | 1.5E+08 | 0.0048 | 0.7641 | 23 | 6.20E-09 | 4.10E-09 | 0.7842 | 0.0201 | ZC3H12D |
| chr14 | 32670194 | 32670600 | 2.10E-06 | 0.7641 | 41 | 5.30E-14 | 1.80E-12 | 0.7831 | 0.0190 | RNU6-2 |
| chr14 | 53618809 | 53619207 | 9.00E-06 | 0.7639 | 39 | 7.90E-13 | 7.80E-12 | 0.7820 | 0.0182 | LOC101927620 |
| chr1 | 1.52E+08 | 1.52E+08 | 0.0024 | 0.7521 | 25 | 1.30E-09 | 2.10E-09 | 0.9977 | 0.2456 | TUFT1 |
| chr3 | 1.8E+08 | 1.8E+08 | 5.10E-14 | 0.7376 | 90 | 4.60E-14 | 4.40E-20 | 0.8305 | 0.0929 | CCDC39 |
| chr2 | 73114227 | 73114548 | 7.20E-10 | 0.7285 | 51 | 4.30E-14 | 6.20E-16 | 0.7590 | 0.0305 | SPR |
| chr12 | 1.07E+08 | 1.07E+08 | 5.50E-11 | 0.6995 | 49 | 4.30E-14 | 4.70E-17 | 0.7020 | 0.0026 | CRY1 |
| chr4 | 2043329 | 2043433 | 0.0057 | 0.6949 | 27 | 6.90E-10 | 4.90E-09 | 0.7823 | 0.0873 | C4orf48 |
| chr11 | 2721866 | 2722358 | 0.00025 | 0.6895 | 29 | 6.20E-11 | 2.20E-10 | 0.9808 | 0.2913 | KCNQ1OT1 |
| chr7 | 27224363 | 27226329 | 8.00E-18 | 0.6825 | 145 | 4.30E-14 | 6.90E-24 | 0.9308 | 0.3326 | HOXA11-AS |
| chr7 | 27224363 | 27226329 | 8.00E-18 | 0.6825 | 145 | 4.30E-14 | 6.90E-24 | 0.9308 | 0.3326 | HOXA11 |
| chr5 | 1.76E+08 | 1.76E+08 | 0.00032 | 0.6782 | 30 | 2.50E-10 | 2.80E-10 | 0.7004 | 0.0222 | SIMC1 |
| chr20 | 47896355 | 47897537 | 0.0058 | 0.6751 | 24 | 2.90E-09 | 5.00E-09 | 0.6992 | 0.0241 | SNORD12B |
| chr20 | 47896355 | 47897537 | 0.0058 | 0.6751 | 24 | 2.90E-09 | 5.00E-09 | 0.6992 | 0.0241 | SNORD12 |
| chr20 | 47896355 | 47897537 | 0.0058 | 0.6751 | 24 | 2.90E-09 | 5.00E-09 | 0.6992 | 0.0241 | ZNFX1 |
| chr19 | 52510955 | 52511839 | 1.00E-13 | 0.6687 | 98 | 4.10E-14 | 8.90E-20 | 0.7648 | 0.0961 | ZNF615 |
| chr5 | 37371443 | 37371686 | 2.80E-05 | 0.6494 | 30 | 2.90E-11 | 2.40E-11 | 0.8208 | 0.1714 | NUP155 |
| chr2 | 1.49E+08 | 1.49E+08 | 0.0031 | 0.6405 | 26 | 6.20E-10 | 2.60E-09 | 0.6430 | 0.0025 | ACVR2A |
| chr5 | 1.13E+08 | 1.13E+08 | 0.0002 | 0.6232 | 37 | 1.00E-12 | 1.70E-10 | 0.6457 | 0.0225 | MCC |
| chr19 | 37157284 | 37158076 | 2.10E-17 | 0.6110 | 76 | 4.20E-14 | 1.80E-23 | 0.6222 | 0.0112 | ZNF461 |
| chr5 | 1.5E+08 | 1.5E+08 | 4.60E-07 | 0.6064 | 40 | 5.70E-14 | 3.90E-13 | 0.9918 | 0.3854 | ZNF300 |
| chr5 | 1.41E+08 | 1.41E+08 | 2.50E-13 | 0.6059 | 71 | 4.10E-14 | 2.10E-19 | 0.6328 | 0.0269 | PCDHGA10 |
| chr11 | 62104932 | 62105023 | 0.00075 | 0.5809 | 29 | 9.40E-10 | 6.50E-10 | 0.9560 | 0.3751 | ASRGL1 |
| chr12 | 9799858 | 9800584 | 2.70E-06 | 0.5693 | 35 | 5.70E-12 | 2.30E-12 | 0.6009 | 0.0317 | LOC374443 |
| chr8 | 30601621 | 30601873 | 7.50E-06 | 0.5579 | 34 | 1.70E-12 | 6.50E-12 | 0.5635 | 0.0056 | UBXN8 |
| chr20 | 57463925 | 57464111 | 2.00E-07 | 0.5037 | 36 | 3.40E-13 | 1.70E-13 | 0.5378 | 0.0340 | GNAS |
| chr20 | 57463925 | 57464111 | 2.00E-07 | 0.5037 | 36 | 3.40E-13 | 1.70E-13 | 0.5378 | 0.0340 | LOC101927932 |
| chr3 | 49314127 | 49314691 | 8.80E-07 | 0.4906 | 52 | 4.50E-14 | 7.50E-13 | 0.9518 | 0.4612 | C3orf62 |
| chr17 | 40610675 | 40611094 | 1.50E-05 | 0.4683 | 45 | 4.20E-14 | 1.30E-11 | 0.5103 | 0.0420 | ATP6V0A1 |
| chr1 | 33546749 | 33546944 | 0.00081 | 0.4582 | 44 | 1.80E-11 | 7.00E-10 | 0.8746 | 0.4164 | AZIN2 |
| chr2 | 1.77E+08 | 1.77E+08 | 0.00017 | 0.4573 | 31 | 8.20E-11 | 1.40E-10 | 0.9761 | 0.5188 | HOXD13 |
| chr13 | 67804060 | 67804406 | 5.50E-05 | 0.4411 | 29 | 6.20E-11 | 4.70E-11 | 0.4425 | 0.0014 | PCDH9 |
| chr11 | 1.13E+08 | 1.13E+08 | 2.30E-05 | 0.4313 | 30 | 2.90E-11 | 1.90E-11 | 0.9746 | 0.5433 | TTC12 |
| chr7 | 1.13E+08 | 1.13E+08 | 8.70E-07 | 0.4288 | 38 | 1.10E-13 | 7.50E-13 | 0.4313 | 0.0025 | LINC00998 |
| chr2 | 1.77E+08 | 1.77E+08 | 2.90E-15 | 0.4245 | 83 | 4.40E-14 | 2.50E-21 | 0.9850 | 0.5604 | HOXD11 |
| chr8 | 37605283 | 37605718 | 2.70E-05 | 0.4132 | 48 | 4.80E-14 | 2.30E-11 | 0.9436 | 0.5303 | LOC728024 |
| chr2 | 1.21E+08 | 1.21E+08 | 8.70E-07 | 0.3748 | 42 | 8.80E-14 | 7.50E-13 | 0.9890 | 0.6141 | TMEM185B |
| chr19 | 52489779 | 52490340 | 4.90E-06 | 0.3544 | 41 | 1.50E-13 | 4.20E-12 | 0.3679 | 0.0135 | ZNF350 |
| chr5 | 1.79E+08 | 1.79E+08 | 2.20E-08 | 0.3466 | 58 | 4.10E-14 | 1.90E-14 | 0.9709 | 0.6243 | RUFY1 |
| chr10 | 52499530 | 52499768 | 0.00068 | 0.3138 | 39 | 2.60E-10 | 5.90E-10 | 0.8940 | 0.5802 | ASAH2B |
| chr19 | 48774927 | 48775128 | 0.0078 | 0.3036 | 28 | 2.80E-10 | 6.70E-09 | 0.3080 | 0.0045 | ZNF114 |
| chr1 | 40420210 | 40420606 | 0.0017 | 0.8422 | 32 | 7.40E-11 | 1.50E-09 | 0.9114 | 0.0692 | MFSD2A |
| chr5 | 1.14E+08 | 1.14E+08 | 5.80E-12 | 0.8370 | 55 | 4.00E-14 | 5.00E-18 | 0.9788 | 0.1418 | KCNN2 |
| chr8 | 41754725 | 41754872 | 0.00017 | 0.8355 | 36 | 4.50E-13 | 1.50E-10 | 0.8959 | 0.0605 | ANK1 |
| chr2 | 1.72E+08 | 1.72E+08 | 0.00067 | 0.8293 | 29 | 9.40E-11 | 5.70E-10 | 0.8502 | 0.0209 | GORASP2 |
| chr3 | 1.71E+08 | 1.71E+08 | 2.00E-05 | 0.8257 | 37 | 2.30E-13 | 1.70E-11 | 0.8380 | 0.0123 | TNIK |
| chr10 | 21814277 | 21814455 | 1.30E-05 | 0.8208 | 32 | 6.30E-12 | 1.20E-11 | 0.8328 | 0.0120 | SKIDA1 |
| chr15 | 45670382 | 45671360 | 1.30E-13 | 0.8180 | 72 | 4.00E-14 | 1.20E-19 | 0.8607 | 0.0427 | GATM |
| chr19 | 54024476 | 54024840 | 0.00076 | 0.8146 | 31 | 1.30E-11 | 6.50E-10 | 0.8521 | 0.0375 | ZNF331 |
| chr19 | 49250180 | 49250616 | 0.0022 | 0.8049 | 24 | 2.90E-09 | 1.90E-09 | 0.8276 | 0.0228 | IZUMO1 |
| chr3 | 1.96E+08 | 1.96E+08 | 4.00E-05 | 0.8004 | 36 | 4.90E-13 | 3.40E-11 | 0.8656 | 0.0652 | TM4SF19 |
| chr3 | 1.96E+08 | 1.96E+08 | 4.00E-05 | 0.8004 | 36 | 4.90E-13 | 3.40E-11 | 0.8656 | 0.0652 | TM4SF19-TCTEX1D2 |
| chr19 | 44645378 | 44646069 | 4.00E-20 | 0.7998 | 92 | 3.70E-14 | 3.50E-26 | 0.8295 | 0.0297 | ZNF234 |
| chr19 | 58951522 | 58951896 | 1.30E-08 | 0.7952 | 48 | 4.50E-14 | 1.10E-14 | 0.9464 | 0.1512 | ZNF132 |
| chr6 | 10419399 | 10419492 | 0.0058 | 0.7890 | 24 | 2.90E-09 | 5.00E-09 | 0.9468 | 0.1577 | TFAP2A |
| chr1 | 2.44E+08 | 2.44E+08 | 3.50E-07 | 0.7791 | 38 | 2.40E-13 | 3.00E-13 | 0.9305 | 0.1515 | ZBTB18 |
| chr6 | 1.26E+08 | 1.26E+08 | 8.40E-06 | 0.7765 | 38 | 1.10E-13 | 7.20E-12 | 0.9752 | 0.1987 | HEY2 |
| chr3 | 44903188 | 44903463 | 2.30E-07 | 0.7713 | 37 | 1.80E-13 | 2.00E-13 | 0.7826 | 0.0113 | MIR564 |
| chr3 | 44903188 | 44903463 | 2.30E-07 | 0.7713 | 37 | 1.80E-13 | 2.00E-13 | 0.7826 | 0.0113 | TMEM42 |
| chr19 | 52391010 | 52391481 | 3.10E-14 | 0.7700 | 65 | 4.30E-14 | 2.60E-20 | 0.7790 | 0.0090 | ZNF577 |
| chr19 | 52391010 | 52391481 | 3.10E-14 | 0.7700 | 65 | 4.30E-14 | 2.60E-20 | 0.7790 | 0.0090 | ZNF649-AS1 |
| chr17 | 47072788 | 47074061 | 1.10E-09 | 0.7681 | 60 | 4.30E-14 | 9.80E-16 | 0.8285 | 0.0604 | IGF2BP1 |
| chr6 | 1.5E+08 | 1.5E+08 | 0.0048 | 0.7641 | 23 | 6.20E-09 | 4.10E-09 | 0.7842 | 0.0201 | ZC3H12D |
| chr14 | 32670194 | 32670600 | 2.10E-06 | 0.7641 | 41 | 5.30E-14 | 1.80E-12 | 0.7831 | 0.0190 | RNU6-2 |
| chr14 | 53618809 | 53619207 | 9.00E-06 | 0.7639 | 39 | 7.90E-13 | 7.80E-12 | 0.7820 | 0.0182 | LOC101927620 |
| chr1 | 1.52E+08 | 1.52E+08 | 0.0024 | 0.7521 | 25 | 1.30E-09 | 2.10E-09 | 0.9977 | 0.2456 | TUFT1 |
| chr3 | 1.8E+08 | 1.8E+08 | 5.10E-14 | 0.7376 | 90 | 4.60E-14 | 4.40E-20 | 0.8305 | 0.0929 | CCDC39 |
| chr2 | 73114227 | 73114548 | 7.20E-10 | 0.7285 | 51 | 4.30E-14 | 6.20E-16 | 0.7590 | 0.0305 | SPR |
| chr12 | 1.07E+08 | 1.07E+08 | 5.50E-11 | 0.6995 | 49 | 4.30E-14 | 4.70E-17 | 0.7020 | 0.0026 | CRY1 |
| chr4 | 2043329 | 2043433 | 0.0057 | 0.6949 | 27 | 6.90E-10 | 4.90E-09 | 0.7823 | 0.0873 | C4orf48 |
| chr4 | 2043329 | 2043433 | 0.0057 | 0.6949 | 27 | 6.90E-10 | 4.90E-09 | 0.7823 | 0.0873 | C4orf48 |
| chr11 | 2721866 | 2722358 | 0.00025 | 0.6895 | 29 | 6.20E-11 | 2.20E-10 | 0.9808 | 0.2913 | KCNQ1OT1 |
| chr7 | 27224363 | 27226329 | 8.00E-18 | 0.6825 | 145 | 4.30E-14 | 6.90E-24 | 0.9308 | 0.3326 | HOXA11-AS |
| chr7 | 27224363 | 27226329 | 8.00E-18 | 0.6825 | 145 | 4.30E-14 | 6.90E-24 | 0.9308 | 0.3326 | HOXA11 |
| chr5 | 1.76E+08 | 1.76E+08 | 0.00032 | 0.6782 | 30 | 2.50E-10 | 2.80E-10 | 0.7004 | 0.0222 | SIMC1 |
| chr20 | 47896355 | 47897537 | 0.0058 | 0.6751 | 24 | 2.90E-09 | 5.00E-09 | 0.6992 | 0.0241 | SNORD12B |
| chr20 | 47896355 | 47897537 | 0.0058 | 0.6751 | 24 | 2.90E-09 | 5.00E-09 | 0.6992 | 0.0241 | SNORD12 |
| chr20 | 47896355 | 47897537 | 0.0058 | 0.6751 | 24 | 2.90E-09 | 5.00E-09 | 0.6992 | 0.0241 | ZNFX1 |
| chr19 | 52510955 | 52511839 | 1.00E-13 | 0.6687 | 98 | 4.10E-14 | 8.90E-20 | 0.7648 | 0.0961 | ZNF615 |
| chr5 | 37371443 | 37371686 | 2.80E-05 | 0.6494 | 30 | 2.90E-11 | 2.40E-11 | 0.8208 | 0.1714 | NUP155 |
| chr2 | 1.49E+08 | 1.49E+08 | 0.0031 | 0.6405 | 26 | 6.20E-10 | 2.60E-09 | 0.6430 | 0.0025 | ACVR2A |
| chr5 | 1.13E+08 | 1.13E+08 | 0.0002 | 0.6232 | 37 | 1.00E-12 | 1.70E-10 | 0.6457 | 0.0225 | MCC |
| chr19 | 37157284 | 37158076 | 2.10E-17 | 0.6110 | 76 | 4.20E-14 | 1.80E-23 | 0.6222 | 0.0112 | ZNF461 |
| chr5 | 1.5E+08 | 1.5E+08 | 4.60E-07 | 0.6064 | 40 | 5.70E-14 | 3.90E-13 | 0.9918 | 0.3854 | ZNF300 |
| chr5 | 1.41E+08 | 1.41E+08 | 2.50E-13 | 0.6059 | 71 | 4.10E-14 | 2.10E-19 | 0.6328 | 0.0269 | PCDHGA10 |
| chr11 | 62104932 | 62105023 | 0.00075 | 0.5809 | 29 | 9.40E-10 | 6.50E-10 | 0.9560 | 0.3751 | ASRGL1 |
| chr12 | 9799858 | 9800584 | 2.70E-06 | 0.5693 | 35 | 5.70E-12 | 2.30E-12 | 0.6009 | 0.0317 | LOC374443 |
| chr20 | 57463925 | 57464111 | 2.00E-07 | 0.5037 | 36 | 3.40E-13 | 1.70E-13 | 0.5378 | 0.0340 | GNAS |
| chr20 | 57463925 | 57464111 | 2.00E-07 | 0.5037 | 36 | 3.40E-13 | 1.70E-13 | 0.5378 | 0.0340 | LOC101927932 |
| chr3 | 49314127 | 49314691 | 8.80E-07 | 0.4906 | 52 | 4.50E-14 | 7.50E-13 | 0.9518 | 0.4612 | C3orf62 |
| chr17 | 40610675 | 40611094 | 1.50E-05 | 0.4683 | 45 | 4.20E-14 | 1.30E-11 | 0.5103 | 0.0420 | ATP6V0A1 |
| chr1 | 33546749 | 33546944 | 0.00081 | 0.4582 | 44 | 1.80E-11 | 7.00E-10 | 0.8746 | 0.4164 | AZIN2 |
| chr2 | 1.77E+08 | 1.77E+08 | 0.00017 | 0.4573 | 31 | 8.20E-11 | 1.40E-10 | 0.9761 | 0.5188 | HOXD13 |
| chr13 | 67804060 | 67804406 | 5.50E-05 | 0.4411 | 29 | 6.20E-11 | 4.70E-11 | 0.4425 | 0.0014 | PCDH9 |
| chr11 | 1.13E+08 | 1.13E+08 | 2.30E-05 | 0.4313 | 30 | 2.90E-11 | 1.90E-11 | 0.9746 | 0.5433 | TTC12 |
| chr7 | 1.13E+08 | 1.13E+08 | 8.70E-07 | 0.4288 | 38 | 1.10E-13 | 7.50E-13 | 0.4313 | 0.0025 | LINC00998 |
| chr2 | 1.77E+08 | 1.77E+08 | 2.90E-15 | 0.4245 | 83 | 4.40E-14 | 2.50E-21 | 0.9850 | 0.5604 | HOXD11 |
| chr8 | 37605283 | 37605718 | 2.70E-05 | 0.4132 | 48 | 4.80E-14 | 2.30E-11 | 0.9436 | 0.5303 | LOC728024 |
| chr2 | 1.21E+08 | 1.21E+08 | 8.70E-07 | 0.3748 | 42 | 8.80E-14 | 7.50E-13 | 0.9890 | 0.6141 | TMEM185B |
| chr19 | 52489779 | 52490340 | 4.90E-06 | 0.3544 | 41 | 1.50E-13 | 4.20E-12 | 0.3679 | 0.0135 | ZNF350 |
| chr5 | 1.79E+08 | 1.79E+08 | 2.20E-08 | 0.3466 | 58 | 4.10E-14 | 1.90E-14 | 0.9709 | 0.6243 | RUFY1 |
| chr10 | 52499530 | 52499768 | 0.00068 | 0.3138 | 39 | 2.60E-10 | 5.90E-10 | 0.8940 | 0.5802 | ASAH2B |
| chr19 | 48774927 | 48775128 | 0.0078 | 0.3036 | 28 | 2.80E-10 | 6.70E-09 | 0.3080 | 0.0045 | ZNF114 |

**Additional File 5.**


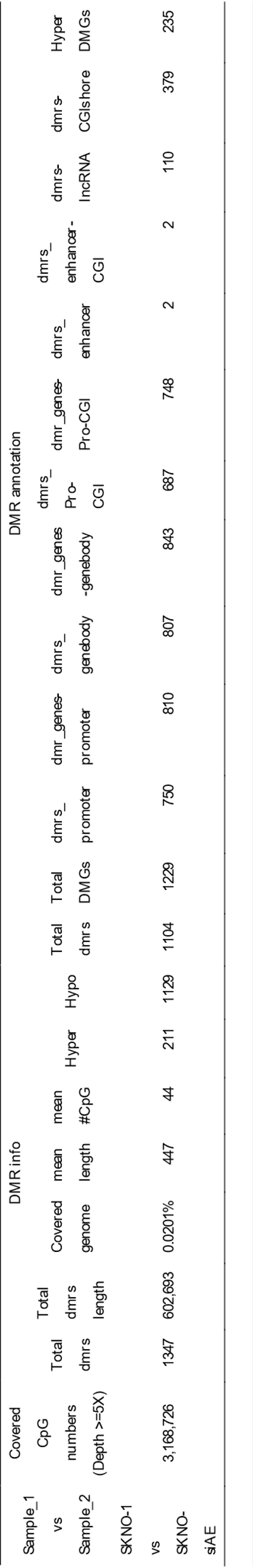

Supplement: Supplementary file 1 — Additional files [file 12276_2021_695_MOESM1_ESM.docx]
